# Barriers and Facilitators to Oral Health Assessment and Referral by Non‐Dental Healthcare Professionals in Primary Health Centres: A Qualitative Study

**DOI:** 10.1155/ijod/3339611

**Published:** 2026-11-01

**Authors:** Amitha Basheer N., Praveen Jodalli, Shishir Ram Shetty, Ashwini Rao, Avinash B. R.

**Affiliations:** ^1^ Department of Public Health Dentistry Manipal College of Dental Sciences Mangalore Manipal Academy of Higher Education Manipal India manipal.edu; ^2^ Department of Oral and Craniofacial Health Sciences College of Dental Medicine University of Sharjah Sharjah UAE sharjah.ac.ae

**Keywords:** early identification, India, non-dental healthcare professionals, oral health screening, primary health centres, qualitative research

## Abstract

**Introduction:**

Non‐dental healthcare professionals (NDHPs) are strategically positioned to support the early identification, prevention and referral of oral health problems within the primary healthcare system. However, oral health assessments are not routinely incorporated into primary health centres (PHCs), particularly in settings without regular dental services.

**Objective:**

This study explored NDHPs’ experiences and perspectives regarding oral health assessment, prevention and referral in PHCs.

**Methods:**

An exploratory qualitative study was conducted among 14 NDHPs selected from one urban and one rural PHC in Mangalore, South India. Participants included community health officers (CHOs), auxiliary nurse midwives (ANMs), and physicians, selected using purposive sampling. Individual face‐to‐face, semi‐structured interviews were conducted. The interviews were audio‐recorded, transcribed verbatim and analysed inductively using Braun and Clarke’s six‐phase thematic analysis approach.

**Results:**

Five major themes emerged: current practices, knowledge gaps, barriers, facilitators, and training and resource needs. Oral health assessment was reported to be largely opportunistic, with limited training, confidence, assessment resources, and referral pathways identified as key challenges. Community trust and willingness to participate in oral health training were important facilitators. The findings informed a preliminary identify‐prevent‐refer (IPR) conceptual framework for integrating oral health into primary healthcare.

**Conclusion:**

Despite gaps in training, resources, and referral pathways, NDHPs demonstrated a willingness to contribute to oral health assessment. The preliminary IPR framework provides a conceptual approach to integrating oral health identification, prevention and referral within the primary healthcare system. Further evaluation across diverse settings is warranted.

## 1. Background

Oral health is an integral component of general health, well‐being and quality of life [1]. It encompasses the health and functioning of the oral cavity, teeth and orofacial structures, which are essential for activities such as eating, breathing and speaking throughout the life course [2]. Oral diseases adversely affect quality of life, social interactions, self‐esteem and overall well‐being [3].

Importantly, oral and systemic health are interconnected through inflammatory, microbial and shared risk pathways. Diabetes mellitus is particularly relevant; oral manifestations reported among individuals with diabetes include periodontal disease, xerostomia, periapical lesions, taste disturbances and mucosal lesions. A systematic review confirmed that these manifestations occurred more frequently among individuals with diabetes [4]. Furthermore, longitudinal evidence has indicated that diabetes is associated with an 86% increased risk of the onset or progression of periodontitis [5]. Conversely, a meta‐analysis reported that diabetes was present in 13.1% of individuals with periodontitis compared with 9.6% of those without periodontitis, demonstrating the bidirectional relationship between oral and systemic health [6]. Therefore, preventive measures and early detection of oral diseases are important for reducing the risk of subsequent complications affecting both oral and overall health.

Periodic assessment of the oral cavity provides an opportunity to identify disease risk, recognise abnormalities at an early stage, provide preventive advice, and initiate appropriate referrals [2]. However, when oral health problems are encountered in non‐dental healthcare settings, especially where access to dental services is limited [7], opportunities for routine oral assessment might be overlooked [8]. In such situations, non‐dental healthcare professionals (NDHPs), such as general practitioners, nurses, midwives and community‐based healthcare workers, are strategically positioned to recognise oral health problems and facilitate timely access to dental services [9]. Their involvement may create additional opportunities for early identification and prevention beyond conventional dental settings.

The integration of oral health into non‐dental care, however, remains constrained by individual‐ and health‐system‐level factors. Limited oral health training, uncertainty regarding professional roles, time constraints, inadequate referral networks and fragmented information systems have been reported as barriers to effective oral health assessment and referral by NDHPs [10–12]. Consequently, NDHPs may provide only temporary relief or symptomatic advice rather than definitive dental care, underscoring the need for structured strategies to empower NDHPs to contribute meaningfully to oral healthcare [10]. Inadequate interprofessional communication and limited collaboration between dental and non‐dental healthcare providers further contribute to fragmented care [10, 11, 13]. In the absence of structured pathways, NDHPs may rely on symptomatic management, general advice or informal referrals rather than systematic assessment and coordinated follow‐up [10–12, 14]. Previous studies have consequently emphasised the need for targeted oral health training, practical screening approaches, clearer referral pathways, improved communication with dental professionals and context‐appropriate strategies such as teledentistry [11–13, 15, 16].

These challenges are particularly relevant to primary healthcare in India, where oral health remains an important but insufficiently integrated component of routine healthcare delivery. NDHPs interact extensively with communities through primary health centres (PHCs), district‐level services and community outreach programmes [17], creating opportunities to incorporate basic oral health assessment and prevention into existing healthcare encounters. Nevertheless, evidence from low‐ and middle‐income settings indicates persistent deficiencies in oral health knowledge, role clarity and referral processes among non‐dental providers [12, 18].

Despite their potential role, there is limited qualitative evidence from Indian PHC settings examining how NDHPs experience and perceive oral health assessment and referral, how individual and organisational factors influence their participation and the support they consider necessary to incorporate these activities into routine practice. This is an important evidence gap because interventions developed without an understanding of these contextual factors might fail to integrate NDHPs effectively into existing primary healthcare workflows.

The present study forms part of a broader research project focused on the development and validation of an oral health assessment tool for NDHPs. As an exploratory component of this project, it was considered necessary to understand existing practices and contextual factors influencing oral health assessment to inform the development of a feasible and contextually appropriate approach. Therefore, this study aimed to explore NDHPs’ experiences and perspectives regarding oral health assessment, prevention and referral in PHC settings, paying particular attention to current practices, perceived preparedness, barriers and enabling factors and the training and resources required to support their involvement.

## 2. Methods

### 2.1. Study Design

An exploratory qualitative study was conducted among NDHPs from two PHCs in Mangalore, South India. Semi‐structured, in‐depth interviews were conducted to explore participants’ experiences, perceptions, current practices, barriers, facilitators and perceived needs related to oral health assessment, prevention and referral within primary healthcare settings. An inductive thematic approach was adopted to allow codes, patterns and themes to emerge from participants’ accounts rather than being based on predetermined analytical categories. The study has been reported in accordance with the Consolidated Criteria for Reporting Qualitative Research (COREQ) Checklist [19].

### 2.2. Study Sites

This study was conducted in two government PHCs located in Mangalore Taluk, Dakshina Kannada district, Karnataka, South India. These facilities were selected in consultation with the district health authorities based on the following inclusion criteria: regular patient flow from the local population, availability of general outpatient services and the absence of a resident dental surgeon at the PHCs. Both PHCs offered general outpatient consultations, vaccination services, maternity, and child healthcare and non‐communicable disease management. Nevertheless, neither PHC offered regular dental services nor had a structured oral health screening procedure.

The rural PHC provided services to geographically dispersed populations with inadequate access to dental care, whereas the urban PHC served a semi‐urban population from a variety of socioeconomic backgrounds. The choice between one rural and one urban location made it possible to explore variations in oral health screening practices across diverse primary care settings.

### 2.3. Study Participants

NDHPs often serve as the first point of contact for healthcare delivery in the community. NDHPs who encountered patients with oral health issues at either the urban or rural PHC in Mangalore, South India, were eligible to participate in the study. Fourteen NDHPs, including four community health officers (CHOs), six auxiliary nurse midwives (ANMs), and four physicians (two general medicine specialists and two MBBS graduates), who provided routine outpatient care, participated in the study. Purposive sampling was used to ensure representation across different professional categories and both urban and rural primary healthcare settings.

Participants were eligible if they were involved in routine patient care, had encountered patients with oral health concerns, and were willing to participate in an individual interview. Dental healthcare professionals, healthcare personnel who were not involved in direct patient care, and those who had not encountered patients with oral health concerns were excluded. Fourteen eligible NDHPs were approached, and all agreed to participate; therefore, there were no refusals or withdrawals.

### 2.4. Data Collection

#### 2.4.1. Research Team and Reflexivity

The interviews were conducted by a female dentist and PhD research scholar in public health dentistry who had received training in qualitative research and had prior experience in oral health research. The interviewer had previously visited the selected PHCs in connexion with dental camps; however, no personal relationship had been established with the participants before recruitment. The participants were informed about the interviewer’s professional background and the purpose of the study before the interviews. During the analysis, interpretations were discussed among members of the research team to consider alternative explanations and minimise the influence of a single researcher’s perspective on theme development.

No statistical sample size calculations were performed. Sample adequacy was determined using the principle of data saturation, whereby recruitment continued until additional interviews no longer generated substantively new information, codes, or themes relevant to the study objectives. Previous methodological research has demonstrated that thematic saturation in relatively focused and homogeneous qualitative interview studies may be achieved within ~12 interviews, although the required sample depends on the specificity and information richness of the participants [20].

The adequacy of the sample was assessed concurrently with data collection and preliminary analysis. After each successive interview, emerging codes and concepts were compared with those identified in the preceding interviews. During the later interviews, the principal concepts became increasingly recurrent, and the research team determined that further interviews were unlikely to provide substantively new themes. Additional interviews were conducted to confirm saturation and ensure representation across different professional groups. Recruitment was therefore concluded after 14 interviews when the research team determined that sufficient depth and redundancy had been achieved across the dataset. Participants were identified through the medical officers in charge of the two PHCs and were provided with an information sheet describing the purpose and procedures of the study. All eligible individuals who were approached agreed to participate, and written informed consent was obtained before the interviews.

The interview guide was developed based on the literature and insights from two researchers who had been actively involved in the study since its inception [11]. It comprised open‐ended questions designed to explore the participants’ perspectives and experiences in greater depth. Face‐to‐face individual interviews were conducted in a private room at each PHC to ensure privacy and allow participants to express their views freely. The interviews were conducted in English and lasted between 5 and 15 min.

All the interviews were audio‐recorded using a digital voice recorder, and the recordings were securely stored on password‐protected computers accessible only to the research team. To minimise social desirability bias, participants were informed that their responses would remain confidential and that they could express their views freely without any fear of consequences. Written informed consent was obtained from all participants before the interviews. At the conclusion of each interview, the interviewer summarised the key points discussed to confirm her understanding of the participants’ responses. Demographic information, including age, sex and professional experience, was also collected. No repeat interviews were conducted, and the transcripts were not returned to the participants for correction or comment. No field notes were recorded during or immediately after the interviews.

The interview guide addressed participants’ roles and responsibilities in PHCs, their experience with oral health screening, challenges in oral health screening, confidence in oral health assessment, availability of screening tools, perceptions of the relationship between oral health and general health, the emphasis placed on oral health in training programmes for non‐dental health professionals, perceived needs for oral health training and resources and their views on potential strategies to improve oral health assessment and referral in the PHC setting. The main questions in the interview guide are as follows:

• Can you brief about your current duties or role as a healthcare professional in this PHC?
• Have you ever conducted any oral health screening or assessment for your patients?
• What are the challenges or difficulties you faced while doing the oral examination/screening?
• Are you confident in doing an oral health assessment?
• Are you employing any screening tools, guidelines or resources for the evaluation of oral health?
• How important do you think oral health is to overall well‐being and general health?
• Do you think that oral health has received sufficient emphasis in the regular training given to non‐dental health professionals?
• What kind of oral health training or resources do you think are lacking in these programmes?
• What can you do to make an oral health assessment relatively easy and feasible?
• If you require training, which aspect of oral health would you prefer to be covered?
• Have you ever collaborated or communicated with a dentist/dental professional about your patients’ oral health issues?
• Do you have any further comments on your oral health evaluation experiences or challenges that we have not covered in this interview?

### 2.5. Data Analysis and Conceptual Synthesis

The interviews were transcribed verbatim into Microsoft Word, and the authors (Amitha Basheer N. and Praveen Jodalli) cross‐checked the transcripts against the audio recordings to ensure their accuracy. Each participant was assigned a numerical code to maintain confidentiality. The transcripts were read repeatedly to achieve familiarity with the data and to understand the participants’ responses within their broader context.

An inductive thematic analysis was conducted following the six‐phase approach described by Braun and Clarke [21]. The analysis began with familiarisation with the transcripts, followed by the generation of initial codes and the development of candidate themes. The themes were reviewed, defined, and named before the final thematic account was produced [21]. Amitha Basheer N. and Praveen Jodalli independently coded the transcripts and identified recurring patterns within the dataset. No predefined coding framework or predetermined thematic categories were imposed on the dataset. The codes were compared across transcripts, and conceptually related codes were grouped into categories and subthemes. These were further developed into overarching themes that reflected recurring patterns in the dataset. The researchers discussed coding differences and interpretations during regular research team meetings and reached consensus on the final thematic structure. To minimise the risk of deductive disclosure, quotations were attributed using unique participant codes (participant 01–participant 14) without reporting the exact ages, genders, study sites or specific professional designations. ATLAS.ti Version 23 for Windows was used to organise the data and support the coding and thematic analysis [22]. All the audio recordings, transcripts and analytical data were securely stored on password‐protected computers accessible only to the research team.

Following thematic analysis, an interpretive conceptual synthesis was undertaken to examine the relationships among the identified themes and their implications for integrating oral health into primary healthcare. The synthesis was based on participants’ accounts of current practices, knowledge gaps, barriers, facilitators and training and resource needs. The findings were organised into three interrelated functions for NDHPs within primary healthcare: identification of oral health problems, provision of preventive oral health guidance and referral to appropriate dental services.

This synthesis informed the development of the identify‐prevent‐refer (IPR) conceptual framework. The framework was subsequently organised across the provider, institutional, community and health system levels to illustrate the contextual and system‐level factors that may support the integration of oral health assessment, prevention and referral into primary healthcare practice.

### 2.6. Ethics Considerations

This qualitative study formed part of a larger research project focused on the development and validation of an OHAT for NDHPs to facilitate the identification of oral health problems and appropriate referrals. The present qualitative phase was undertaken to understand existing oral health assessment practices and identify perceived knowledge gaps, barriers, facilitators and training and resource needs among NDHPs. These findings were intended to provide contextual evidence to inform subsequent phases of the broader project, including the development and implementation of strategies to support oral health assessment and referral in primary healthcare settings.

Ethics approval was obtained from the Institutional Ethics Committee prior to the commencement of the study (Reference Number 24024). Participation was entirely voluntary, and written informed consent was obtained from all participants before the interviews. Participants were assured that their responses would remain confidential, and all identifying information was removed from the transcripts and study outputs.

## 3. Results

In total, 14 interviews were conducted. Four of the 14 interviews were conducted with CHOs, six with ANMs, and four with physicians (two general practitioners [MBBS graduates] and two general medicine specialists [masters in general medicine]). Twelve of the 14 participants were female and two were male. The age range of the participants was 25–51 years. The majority of participants had worked at the PHC for more than 5 years. This is presented in Table 1. The five major themes and subthemes that emerged from the interview data are illustrated in Figure 1. Themes, subthemes, categories and codes derived from the analysis of the interviews are presented in Table 2.

**Figure 1:**
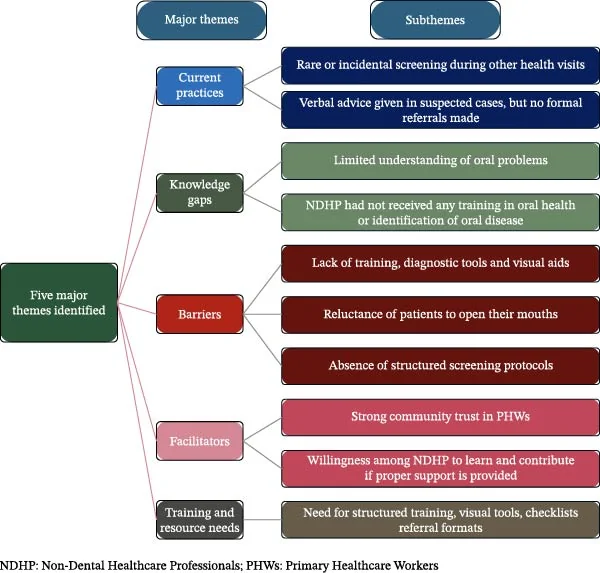
Common themes and subthemes. NDHP, non‐dental healthcare professionals; PHWs, primary healthcare workers.

**Table 1:** Characteristics of the participants.

Characteristic	Number (%)
Gender	
Female	12 (85.7)
Male	2 (14.3)

Age groups (years)	
20–30	4 (28.6)
31–40	5 (35.7)
41–50	4 (28.6)
>50	1 (7.1)

Professional category	
Community health officer (CHO)	4 (28.6)
Auxiliary nurse midwife (ANM)	6 (42.9)
Physician (GP + GM)	4 (28.6)

Years of professional experience	
1–5 years	4 (28.6)
6–10 years	7 (50.0)
>10 years	3 (21.4)

*Note:* The data are presented as frequencies with percentages in parentheses.

**Table 2:** Themes derived from the analysis of the interviews.

Theme	Subtheme	Categories	Codes
Current practices	Oral health screening practices	Screening approach	Incidental oral examination; symptom‐based examination; opportunistic screening; oral examination only when complaints are present
	Oral health advice	Advice provided	Oral hygiene advice; tobacco reduction advice; reassurance; advice to seek dental consultation
	Referral practices	Referral approach	Informal dental referral: verbal recommendation to visit dentist; absence of formal referral pathway; limited follow‐up
Knowledge gaps	Knowledge of oral health conditions	Recognition of common problems	Recognition of toothache; oral ulcers; gum problems; swelling
		Recognition of serious conditions	Difficulty differentiating minor and serious lesions; uncertainty in recognising early oral disease; limited awareness of suspicious lesions
	Oral health education and training	Previous training	Limited oral health content in professional education; no formal oral screening training; limited training in tobacco‐related lesions; limited oral cancer identification training
Barriers	Professional‐level barriers	Knowledge and skills	Lack of oral health training; limited confidence in oral examination; uncertainty in lesion identification
	Resource‐related barriers	Screening resources	Lack of diagnostic tools; absence of visual aids; lack of screening checklists
	Patient‐related barriers	Patient cooperation	Reluctance to open mouth; discomfort during oral examination
	System‐level barriers	Organisation of services	Absence of screening protocols; oral health not included in routine workflow; competing clinical priorities; lack of structured referral system
Facilitators	Community‐related facilitators	Community relationship	Trust in healthcare workers; NDHPs as first point of contact; established community relationships
	Provider‐related facilitators	Readiness to participate	Willingness to perform oral assessment; willingness to learn; interest in receiving training; willingness to provide oral health guidance
Training and resource needs	Training needs	Knowledge and skill development	Training in oral examination; recognition of common oral diseases; identification of suspicious lesions; oral health counselling
	Screening resources	Practical assessment support	Visual diagnostic guides; oral health screening checklist; simple screening tools
	Referral support	Referral and continuity of care	Standard referral format; clear referral criteria; communication with dental professionals; follow‐up mechanisms

### 3.1. Current Practices

All participants reported that oral health screening was not routinely conducted as part of standard clinical examinations in PHCs. Oral examinations were usually performed incidentally during other health consultations, particularly when patients presented with visible oral complaints or symptoms such as pain, ulcers or swelling. Participants explained that oral health assessments were rarely included in routine health visits because of competing priorities and the absence of structured screening protocols.

Usually, I check the mouth only if the patient complains of pain or ulcers. Otherwise, I focus on their main health problem…. (participant 01)

This reflected a predominantly symptom‐driven approach to oral health assessment, in which the examination of the oral cavity was prompted by an existing complaint rather than being incorporated into routine clinical assessment. The emphasis on the presenting medical problem also indicated that the oral health assessment was not consistently perceived as part of the standard clinical encounter.

In most cases, participants reported that oral health problems were identified opportunistically rather than through systematic screening. When participants encountered patients with suspected oral health problems, the most common response was to provide basic verbal advice. Participants frequently advised patients to maintain oral hygiene, reduce tobacco use, or seek a dental consultation. However, formal referral systems were rarely used, and follow‐up of such cases was uncommon.

…If I see something unusual in the mouth, I usually advise them to go to a dentist. However, there is no formal system to refer them…. (participant 02).

The absence of a formal referral mechanism meant that the recognition of an oral health problem was not consistently followed by a defined process for referral or follow‐up. Thus, participants’ involvement generally extended to recognising the problem and advising the patient, with limited evidence of a structured pathway linking assessment to subsequent dental care. Several participants noted that they primarily offered general health advice rather than detailed oral health guidance as they felt that their knowledge in this area was limited. Participants also described using informal or basic approaches when dealing with oral complaints. It was also reported that in many situations, healthcare providers focused on providing reassurance or recommending that patients visit a dental facility in nearby hospitals.

…Sometimes patients come with mouth ulcers or gum problems. I usually advise them to maintain oral hygiene and consult a dentist if the problem continues…. (participant 03)

Across these descriptions, oral health assessment, advice, and referrals appeared to function as separate and largely informal activities rather than as components of a consistent care process. The participants’ descriptions therefore indicated that the extent of oral health involvement was shaped by the presence of symptoms and the immediate demands of the consultation.

The findings within this theme indicate a consistent pattern in which oral health assessment was initiated primarily by patient‐reported symptoms or visible abnormalities rather than being incorporated into routine clinical assessment. This symptom‐driven approach was accompanied by a reliance on general advice and informal dental referrals, with limited evidence of structured follow‐up.

### 3.2. Knowledge Gaps

The participants reported limited knowledge regarding oral health conditions and the early identification of oral diseases. Many of the NDHPs stated that oral health had not been adequately covered during their professional education or routine healthcare training programmes. As a result, participants expressed uncertainty in recognising early signs of oral lesions and other oral health problems.

Several participants indicated that they were aware of common oral complaints such as toothache or ulcers but lacked the knowledge to differentiate between minor conditions and potentially serious oral diseases. This distinction between recognising familiar symptoms and interpreting their clinical significance was evident across the interviews. This lack of knowledge often limited their ability to confidently perform oral examinations or provide appropriate guidance to patients.

…I can recognise basic problems such as ulcers or pain, but I am not sure how to identify serious oral diseases…. (participant 04)

This response illustrates that participants’ perceived competence was greater for recognising common oral complaints than for interpreting potentially significant abnormalities. This difficulty was therefore not limited to identifying an oral problem but extended to determining its significance and the appropriate subsequent action.

Many participants also mentioned that they had not received any formal training related to oral health screening, particularly in identifying tobacco‐related lesions or early signs of oral cancer. Consequently, oral health assessments are not routinely incorporated into clinical practice.

…During our training, we were not taught how to examine the mouth properly or identify oral diseases… (participant 05)

The reported lack of formal training was accompanied by uncertainty regarding how to conduct an oral examination and interpret abnormal findings, particularly those requiring further assessment or referral. Participants consistently linked their limited confidence in recognising oral lesions to inadequate educational preparation and the absence of continuing professional training. Consequently, uncertainty in recognising potentially serious oral conditions appeared to reinforce dependence on symptom‐based assessment and informal advice rather than proactive oral health screening and referral.

The findings showed that participants’ difficulties extended beyond recognising common oral conditions to identifying abnormal findings and determining their significance. Limited formal training was frequently accompanied by lower confidence in conducting oral examinations and recognising findings requiring further attention or referral. These patterns were reflected in participants’ descriptions of their routine oral health assessment practices.

### 3.3. Barriers

The participants reported several barriers that limited their ability to conduct oral health assessments within primary healthcare settings. The most frequently mentioned challenges included a lack of training, the absence of diagnostic tools and visual aids, the reluctance of patients to open their mouths during examinations and the absence of structured oral health screening protocols.

Many participants emphasised that they had not received adequate training in oral health assessment, which reduced their confidence in identifying oral diseases. In addition, participants noted that diagnostic tools and visual aids that could assist in recognising oral lesions were not available in their PHCs.

We have not received proper training to identify oral diseases, and there are no tools or visual guides available to help us. (participant 06)

The quotation demonstrates that participants experienced limitations in both their preparation and the practical support available to them during oral examinations. The absence of training and visual resources was described together, indicating that participants lacked both the knowledge and practical aids needed to support the recognition of oral conditions.

Participants also reported that some patients were hesitant or reluctant to open their mouths during the examinations. This reluctance sometimes made it difficult to perform even basic oral inspections, especially during busy outpatient consultations.

….Sometimes patients are not comfortable opening their mouths for examination, so it becomes difficult to check properly…. (participant 07)

Patient reluctance emerged as an additional practical constraint, limiting the extent to which oral examinations could be undertaken during routine consultations. This difficulty was particularly relevant when oral assessment had to be incorporated into already time‐constrained clinical encounters.

Another frequently mentioned barrier was the absence of structured protocols or guidelines for oral health screening in primary healthcare settings. Participants explained that oral health assessment was not included in routine clinical workflows, which limited the opportunities for systematic screening.

There is no specific protocol or guideline for oral health screening in our practice, so I only check when patients complain. (participant 08)

This response illustrated how the absence of a defined screening process left the initiation of oral assessment dependent on the presenting complaints. Rather than following a consistent assessment procedure, participants reported that the decision to examine the oral cavity was determined by the circumstances of the individual consultation.

In the absence of a defined protocol, choices regarding when and how to perform oral assessments were largely dependent on presenting symptoms and individual clinical judgement. Across the interviews, barriers were identified at several levels, including training, availability of practical resources, patient interaction, and the organisation of oral health screening within routine practice. These factors collectively contributed to the predominantly opportunistic approach to oral health assessment described by the participants. The findings therefore showed that barriers to oral health assessment extended across the clinical encounter, from participants’ preparedness and available resources to patient cooperation and the organisation of assessment within routine workflows.

### 3.4. Facilitators

Despite the challenges identified, participants also highlighted several factors that could facilitate the integration of oral health assessment within primary healthcare settings. One of the key facilitators identified was the strong level of trust that community members placed in primary healthcare workers. Participants explained that this trust often encouraged patients to seek advice from them regarding various health concerns, including oral health problems.

…People in the community trust us and usually come to us first when they have any health problem…. (participant 09).

The established trust between PHC workers and community members positioned them as accessible sources of health advice and created an existing point of contact through which oral health concerns could be raised during routine healthcare encounters.

….If we receive proper training and guidance, we would definitely be willing to check and identify oral health problems in our patients…. (participant 10)

The willingness expressed here suggests that participants were receptive to taking on a greater role in oral health assessment when appropriate preparation and guidance were available. The established relationship between PHC workers and community members was perceived as an existing opportunity through which oral health concerns and preventive messages could be addressed during routine healthcare encounters.

In addition, many participants expressed a willingness to contribute to oral health assessments if appropriate support and training were provided. Healthcare workers indicated that with proper guidance, tools, and structured training, they would be more confident in identifying oral health problems and advising patients accordingly.

We are willing to check patients’ mouths if we are given simple training and clear guidance on what to look for… (participant 11)

The established relationship between PHC workers and community members was perceived as an existing opportunity through which oral health concerns and preventive messages could be addressed during routine healthcare encounters. The findings within this theme therefore centred on two complementary facilitators: an established relationship of trust between PHC workers and the community and participants’ readiness to engage in oral health activities when adequate support is provided. These facilitators were described alongside the practical conditions that participants considered necessary for their involvement in oral health assessments.

### 3.5. Training and Resource Needs

The participants consistently emphasised the need for structured training programmes and practical resources to support oral health assessment within primary healthcare settings.

Many participants reported that receiving formal training in oral health screening and identifying common oral diseases could improve their confidence and ability to identify oral health problems during routine patient interactions.

Several participants also highlighted the importance of having simple and practical tools that could assist them in conducting oral examinations. These included visual guides illustrating common oral conditions, screening checklists to guide examinations and clear referral formats that would help facilitate timely referral to dental professionals when necessary.

….If we had proper training and simple visual guides, it would be much easier for me to identify oral health problems…. (participant 12)

The emphasis on both training and visual guides suggests that participants perceived knowledge development and practical examination support as complementary requirements for recognising oral health problems during routine consultations. They further reported that structured screening checklists and referral protocols could help integrate oral health assessments into routine healthcare services without significantly increasing their workload.

….a checklist or guideline would help to remember what to look for during oral examination and when to refer the patient. (participant 13)

The preference for a checklist reflected participants’ need for a clear and practical structure to guide both the examination process and decisions regarding referral, particularly within routine clinical workflows.

If there is a proper referral system, it would be easier for us to send patients to the dentist when we identify a problem. (participant 14)

The emphasis on a formal referral system extended the perceived need for support beyond the examination itself, with participants identifying a referral as a necessary step following the recognition of an oral health problem.

Participants indicated that structured training programmes, visual diagnostic aids, simplified screening tools and well‐defined referral pathways would be essential to support NDHPs in effectively identifying and referring patients with oral health problems. Participants viewed these resources not as isolated requirements but as complementary components of a broader system enabling oral health integration. The pattern across the interviews revealed that participants’ perceived needs extended from developing individual competence to providing practical tools and establishing processes that could support assessment and referral within routine PHC practice.

## 4. Discussion

This qualitative study investigated the experiences, perceptions, barriers, and facilitators related to oral health assessment or screening, oral health prevention, and referral among NDHPs. The findings indicate that although oral health screening was not routinely integrated into PHC practices, participants showed a favourable attitude toward incorporating oral health assessments when adequate training and institutional support were provided.

The accounts from ANM, CHO, GP and GM participants indicate that oral health screening has not been integrated into standard PHC examinations. Examinations were reported to be conducted incidentally when patients presented with visible oral complaints such as pain, ulcers or swelling rather than as part of a structured screening protocol. This finding is consistent with evidence from PHC settings, where preventive oral health activities are deprioritised because of competing healthcare priorities and the lack of standardised screening tools [23–25].

The practice of opportunistic oral examinations and verbal advice in PHCs is consistent with findings from multiple PHC studies highlighting the underutilisation of preventive oral health activities, the absence of validated screening tools and the lack of integrated referral systems in routine care [15, 24–27]. When problems were suspected, the typical response was to provide generic advice on oral hygiene, tobacco reduction, and dental consultation rather than to deliver an in‐depth oral health plan or a structured referral. The absence of formal referral systems and low rates of follow‐up were also reflected in the participant accounts: ‘there is no formal system to refer them’ and ‘follow‐up of such cases was uncommon’. This opportunistic approach was consistent with a broader literature pattern in PHCs where opportunistic screening was common but not systematised and where interprofessional referral pathways and feedback mechanisms were often underdeveloped [15, 24, 26, 28, 29].

Several participants indicated limited knowledge of oral health, preferring general health guidance over specialised oral health counselling. These findings are consistent with wider research that identified gaps in oral health training among non‐dental providers and linked these gaps with reduced use of structured screening tools and referral processes [15, 25, 26, 30]. The literature emphasises that dedicated screening protocols, validated assessment tools and formal referral pathways are critical for consistent practice in PHCs [2, 26, 28, 29, 31]. In the present study, the absence of structured screening protocols and formal referral pathways was associated with the opportunistic nature of oral health screening and infrequent follow‐up [15, 24, 26, 28]. The literature also highlights that the integration of oral health into primary care requires context‐specific adaptation of tools, training and workflows rather than a one‐size‐fits‐all model. This is consistent with variation across PHC settings in terms of resources, staffing and health system governance, highlighting the need for locally tailored interventions [15, 32, 33].

The data revealed that participants were able to recognise basic problems such as ulcers or pain but reported limited confidence and skills in identifying serious oral diseases or precancerous or malignant signs. This finding was consistent with broader findings showing that many non‐dental clinicians felt underprepared to perform oral examinations or recognise red flags for oral cancer or other significant pathologies [24, 34]. Participants reported that oral health was not adequately covered in ongoing professional development, leading to uncertainty in recognising early signs of oral lesions and providing appropriate guidance. This aligned with the findings of Vernon et al. [25], who reported that primary care providers often had inadequate training and limited integration of oral health into routine care, suggesting a need for dedicated curricula and competency standards. Reports of the absence of readily available visual guides or diagnostic aids in PHCs parallel findings that the lack of such tools may undermine confidence and performance in recognising and classifying oral conditions [24, 26]. Reluctance or hesitation among patients to open their mouths during examinations could further complicate basic inspections, a barrier frequently noted in primary care dental screening initiatives and one that may be addressed through patient education and trust‐building strategies [26, 34].

Strong community trust in PHCs may provide an opportunity to introduce oral health education and screening as part of routine care, thereby potentially increasing the uptake and acceptance among patients [23, 26]. Participants’ readiness to engage in oral health training and use structured tools indicates the potential for capacity‐building interventions where appropriate support is available. This finding is consistent with evidence suggesting that targeted training may improve knowledge and practices when combined with supportive infrastructure [25, 30, 34]. The demand for simple, practical tools (visual guides, screening checklists and referral templates) and formal training programmes mirrors evidence in the literature that practical educational interventions paired with user‐friendly tools may enhance non‐dental providers’ screening and referral competence [2, 33].

Knowledge gaps among NDHPs regarding oral health assessment appeared closely interconnected with broader organisational constraints, including limited training opportunities, a lack of readily accessible assessment resources and poorly defined referral pathways. Moreover, participants described several factors that could support greater involvement in oral healthcare, including established community relationships, the willingness to undertake additional training and interest in simple assessment and referral resources. These findings reflect participants’ perceived needs and experiences and cannot be interpreted as evidence of the effectiveness of any specific screening tool, training programme or referral intervention. Rather, they identify areas that warrant further investigation when considering the integration of oral health activities into primary healthcare.

Existing validated oral health assessment instruments provide an important context for interpreting participants’ expressed need for structured assessment support. The oral health assessment tool (OHAT) was developed as an eight‐category screening instrument for use by carers and has demonstrated acceptable validity and reliability in residential care settings [35]. Similarly, the revised oral assessment guide (ROAG) has been evaluated as a prediagnostic oral assessment instrument for use by trained community health workers [36]. A systematic review evaluating oral health assessment instruments for NDHPs identified OHATs and ROAGs as among the most complete instruments, with comparatively favourable evidence regarding their measurement properties [37]. More recently, a modified ROAG for non‐dentists (ndROAG) demonstrated improved validity and reliability and incorporated guidance for self‐care and referral [38]. These findings indicate that structured oral assessment by non‐dental personnel has been examined in specific contexts, while its applicability across different primary healthcare settings remains context‐dependent.

Although the themes are presented separately to facilitate the reporting, they represent interconnected dimensions of the same phenomenon. The participants’ accounts indicate that the opportunistic nature of oral health assessment in PHCs was shaped by the interaction of provider‐level, organisational, and health system factors. Limited oral health knowledge was shown to reduce confidence in recognising oral disease, while the absence of structured assessment resources, referral pathways, and institutional protocols restricted the ability to incorporate oral health into routine practice. Conversely, participants’ willingness to undertake additional responsibilities, together with the established trust placed in PHC providers by the community, suggested that the identified barriers are potentially modifiable through coordinated organisational support rather than individual behavioural change alone. These interrelationships formed the basis for the subsequent interpretive synthesis of the findings into the IPR conceptual framework. Its purpose is to provide a broader implementation perspective by illustrating how oral health assessment, preventive guidance and referral may be integrated into routine primary healthcare within the context of the participants’ experiences.

The framework outlines the role of NDHPs in the early identification of oral health problems, provision of basic preventive advice, and timely referral. The proposed conceptual framework is illustrated in Figure 2. Three interrelated functions emerged from this synthesis: the identification of oral health problems, provision of preventive oral health guidance, and referral to appropriate dental services. The framework was therefore not an a priori theoretical framework used to guide data collection or thematic analysis, nor was it evaluated as an intervention in the present study. Rather, it represents a preliminary conceptual organisation of the relationships identified across the qualitative findings.

**Figure 2:**
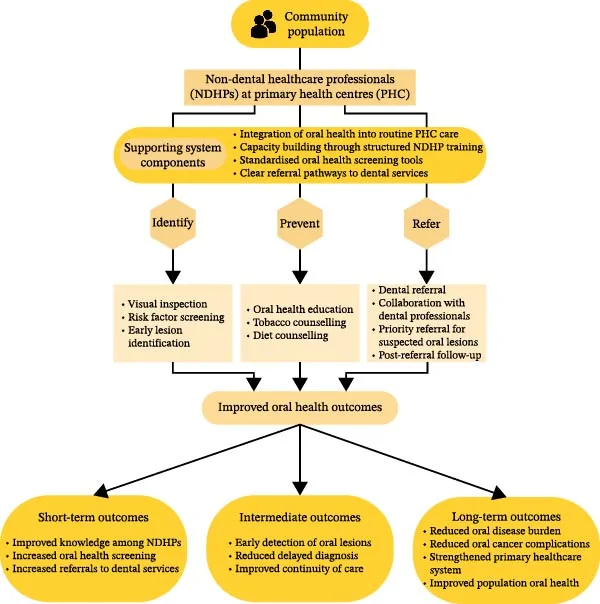
Proposed conceptual framework: identify‐prevent‐refer (IPR) model.

Within the IPR framework, identification reflects participants’ potential role in recognising common oral abnormalities and risk indicators during routine healthcare encounters; prevention encompasses basic oral hygiene, dietary and tobacco‐related guidance; and referral represents the connexion between the identification of a potential problem and appropriate dental evaluation. The findings further suggest that these functions are influenced by factors operating at the provider, institutional, community and broader health system levels, including training, availability of assessment resources, workflow integration, communication with dental professionals and referral mechanisms.

Effective integration of these functions may contribute to improved oral health and reduced disease burden; however, these downstream outcomes were not assessed in the present study. These outcomes therefore represent potential implications of the framework rather than empirical findings. The IPR framework should therefore be regarded as a preliminary hypothesis‐generating conceptual model rather than a validated implementation framework. Further research is needed to refine the framework and evaluate its feasibility, acceptability, applicability and potential impact across diverse primary healthcare settings.

### 4.1. Limitations of the Study

The research was conducted in a limited number of PHCs within a single geographic region, which might restrict the transferability. In addition, the interviews were relatively brief because they were conducted during routine clinical hours, which could have limited the depth and richness of the participants’ accounts. Although information relevant to the study objectives was generated by the focused interview guide, longer or repeat interviews could have facilitated a more nuanced exploration of the participants’ experiences and perspectives. Furthermore, the perspectives of patients were not explored, which could have provided complementary insights into oral health practices and barriers within primary healthcare settings. Future studies involving multiple centres, a more diverse sample and longer qualitative interviews are recommended to enhance the transferability and depth of the findings.

## 5. Conclusion

Oral health assessments were not routinely integrated into the participating PHCs, despite NDHPs recognising opportunities to contribute to oral health identification, preventive guidance and referrals. The findings highlight perceived gaps in preparedness, resources, and referral support, alongside a willingness among NDHPs to participate in oral health activities. The preliminary IPR framework synthesises these findings into three interrelated functions: identification, prevention and referral. It provides a conceptual organisation of the findings that may support the consideration of oral health integration within primary healthcare. However, its feasibility, acceptability, applicability and potential impact require further evaluation across diverse primary healthcare settings.

This study highlights the potential role of NDHPs in the early identification of oral health problems within the primary healthcare setting. However, oral health assessments are not routinely integrated into current practice and are often performed only when patients present with visible symptoms or complaints. Limited training, a lack of diagnostic tools and visual aids, and the absence of structured screening protocols were identified as key barriers to conducting oral health assessments in PHCs.

Despite these challenges, strong community trust in primary healthcare workers and their willingness to participate in oral health activities represent important facilitators that may support greater integration of oral health into the primary care setting. Structured training, visual diagnostic aids, screening checklists and clear referral pathways may provide practical support for NDHPs in identifying and referring patients with oral health problems. These components warrant further investigation as potential mechanisms for strengthening oral health integration into primary healthcare services.

## Author Contributions

Amitha Basheer N. contributed to conceptualisation, data curation, formal analysis, investigation, methodology, writing – original draft and writing – review and editing. Praveen Jodalli contributed to conceptualisation, data curation, methodology, project administration, supervision, formal analysis and writing – review and editing. Shishir Ram Shetty and Avinash B. R. contributed to conceptualisation, methodology, writing – original draft and writing – review and editing. Ashwini Rao contributed to conceptualisation, methodology, resources, writing – original draft and writing – review and editing. All the authors have made substantial contributions to the conception and design of the study, acquisition of data or analysis and interpretation of data and have draughted the article or revised it critically for important intellectual content.

## Funding

This study did not receive any specific funding.

## Disclosure

After using EnglishEDIT.AI, the contents were carefully reviewed and validated by the authors. The authors remain fully responsible for the accuracy, integrity and originality of the content presented in this manuscript. All the authors have read and approved the final version of the manuscript. Praveen Jodalli, as the guarantor of the manuscript, had full access to all the data generated in this study and takes full responsibility for the integrity of the data and the accuracy of the data analysis.

## Conflicts of Interest

The authors declare no conflicts of interest.

## Supporting Information

Additional supporting information can be found online in the Supporting Information section.

## Supporting information


**Supporting Information** COREQ (Consolidated Criteria for Reporting Qualitative Research) checklist.

## Data Availability

The authors confirm that the data supporting the findings of this study are available within the article and/or its Supporting Information.
